# Hyperpolarization and the physical boundary of Liouville space

**DOI:** 10.5194/mr-2-395-2021

**Published:** 2021-06-08

**Authors:** Malcolm H. Levitt, Christian Bengs

**Affiliations:** School of Chemistry, University of Southampton, SO17 1BJ, Southampton, UK

## Abstract

The quantum state of a spin ensemble is described by a density operator, which corresponds to a point in the Liouville space of orthogonal spin operators. Valid density operators are confined to a particular region of Liouville space, which we call the physical region and which is bounded by multidimensional figures called simplexes. Each vertex of a simplex corresponds to a pure-state density operator. We provide examples for spins I=1/2 , I=1 , I=3/2 and for coupled pairs of spins-1/2. We use the von Neumann entropy as a criterion for hyperpolarization. It is shown that the inhomogeneous master equation for spin dynamics leads to non-physical results in some cases, a problem that may be avoided by using the Lindbladian master equation.

## 1 Introduction

The central object of interest in nuclear magnetic resonance (NMR) theory is the density operator, which describes the quantum state of the ensemble of spin systems. It is defined as follows:

1 $$\rho =\bar{|\psi \rangle \langle \psi |}.$$

Here |ψ〉 specifies the quantum state of each individual spin system, and the overbar indicates an ensemble average [Bibr bib1.bibx34]. When expressed as a matrix in the eigenbasis of the coherent spin Hamiltonian, the diagonal elements are the spin state populations, and the off-diagonal elements are the coherences between the spin states.

It is often useful to express the density operator as a superposition of orthogonal spin operators. For example, the highly influential papers by Sørensen, Bodenhausen, Ernst and co-workers advocate an expansion in terms of Cartesian product operators [Bibr bib1.bibx69], while some other groups favour spherical tensor operators [Bibr bib1.bibx62]. In all cases, the density operator is written in the form

2 $$\rho =\sum_{q=1}^{N_{L}}\rho_{q}Q_{q},$$

where the coefficients $\rho_{q}$ are complex numbers in general, and the basis operators $Q_{q}$ are orthogonal:

3 $$\left(Q_{q}|Q_{q^{\prime }}\right)=\text{Tr}\{Q_{q}^{†}Q_{q^{\prime }}\}=\delta_{qq^{\prime }}\|Q_{q}{\|}^{2}.$$

The Kronecker delta symbol $\delta_{ab}$ takes the value 1 for a=b and 0 otherwise. The norm of the operator $Q_{q}$ is defined as $\left\|Q_{q}\|=\text{Tr}\{Q_{q}^{†}{Q_{q}}^{1/2}\right\}$ .

The coefficients $\rho_{q}$ are often given evocative names which suggest their physical interpretation, for example “antiphase order”, “ zz order”, “spin alignment”, “Zeeman polarization”, ”singlet order”, and so on.

Since such expansions are nearly universal in modern NMR theory, it seems natural to pose questions of the forms “what values may the coefficients $\rho_{q}$ take?”, “are the values of $\rho_{q}$ unlimited, or bounded in some way?”, “does the value of one coefficient influence the possible values of a second coefficient?”, etc. Surprisingly, these natural questions are rarely posed in the NMR world, although they have not escaped the attention of mathematical physicists and applied mathematicians [Bibr bib1.bibx25].

The expansion in Eq. ([Disp-formula Ch1.E2]) identifies the density operator with a point in a multidimensional space with coordinates $\left\{\rho_{1},\rho_{2}\ldots \right\}$ . This space has been called *Liouville space* [Bibr bib1.bibx11]. In this article, we show that valid density operators may only be identified with points in a defined region of Liouville space which we call the *physical region*. The physical region is enclosed by a convex boundary, which we call the *physical boundary of Liouville space*. We ask: what is the shape of the physical boundary? Does it have straight edges, or is it spherical in all dimensions? (Spoiler: at least some edges are straight.)

In addition, we express some views on the nature and definition of hyperpolarization. For example, is pure parahydrogen hyperpolarized, even though it generates no NMR signal? (Spoiler: our answer is yes.)

## 2 Orthonormal operators

To facilitate the discussion, the basis operators $Q_{q}$ in Eq. ([Disp-formula Ch1.E2]) are henceforth considered to be normalized as well as orthogonal, so that Eq. ([Disp-formula Ch1.E3]) is replaced by the simpler form

4 $$\left(Q_{q}|Q_{q^{\prime }}\right)=\text{Tr}\{Q_{q}^{†}Q_{q^{\prime }}\}=\delta_{qq^{\prime }}.$$

Note, however, that the Cartesian product operators advocated by [Bibr bib1.bibx69] are *not* normalized.

In general, $N_{L}$ operators are required in the expansion of Eq. ([Disp-formula Ch1.E2]), where $N_{L}=N_{H}^{2}$ and $N_{H}$ is the dimension of the Hilbert space of the individual spin systems. The orthonormal operators $\left\{Q_{1},Q_{2}\ldots Q_{N_{L}}\right\}$ define a $N_{L}$ -dimensional Liouville space [Bibr bib1.bibx42]. The density operator may be represented as a point with coordinates $\left\{q_{1},q_{2}\ldots q_{N_{L}}\right\}$ in this space. All spin dynamics may be represented as a trajectory traced by the spin density operator as it moves through this abstract space.

Brief consideration shows that there must be limits to the physical region of Liouville space. Consider for example an ensemble of isolated spins-1/2. In this case, the dimension of Hilbert space is $N_{H}=2$ , and the dimension of Liouville space is $N_{L}=4$ . The following four normalized operators may be chosen as the basis of Liouville space:

5 $$\begin{array}{rl} Q_{1}=2^{-1/2}I\;\;\; & Q_{2}=2^{1/2}I_{x}\\ Q_{3}=2^{1/2}I_{y}\;\;\; & Q_{4}=2^{1/2}I_{z} \end{array}$$

Since Tr{ρ}=1 by definition, the first coefficient is fixed at $q_{1}=2^{-1/2}$ , the density operator is only free to move in the subspace formed by the other three operators, $\left\{Q_{2},Q_{3},Q_{4}\right\}$ , which are proportional to the angular momentum operators in the three Cartesian directions.

The populations of the two Zeeman states are given by

6 $$\begin{array}{rl} \langle \alpha |\rho |\alpha \rangle & =2^{-1/2}(q_{1}+q_{4})\\ \langle \beta |\rho |\beta \rangle & =2^{-1/2}(q_{1}-q_{4}) \end{array}$$

Both state populations are, by definition, bounded by 0 and 1 :

7 $$\begin{array}{r} 0\leq \langle \alpha |\rho |\alpha \rangle \leq 1\\ 0\leq \langle \beta |\rho |\beta \rangle \leq 1 \end{array}$$

Hence the coefficient $q_{4}$ is bounded as follows:

8 $$-2^{-1/2}\leq q_{4}\leq 2^{-1/2}.$$

The upper bound $q_{4}=2^{-1/2}$ corresponds to maximum spin polarization along the positive z axis, with the |α〉 state completely populated and the |β〉 state completely depleted. The lower bound $q_{4}=-2^{-1/2}$ corresponds to maximum spin polarization along the negative z axis, with the |α〉 state completely depleted and the |β〉 state completely populated.

In the case of isolated spins-1/2, the physical bounds on Liouville space are therefore defined by the fixing of one coordinate ( $q_{1}=2^{-1/2}$ ) and the constraint of the other three to the interior of a sphere of radius $2^{-1/2}$ . Within a numerical factor, this geometrical bound is of course identical to the familiar Bloch sphere – the seminal geometrical object in magnetic resonance theory.

What about systems other than spins-1/2? Liouville space has more than three dimensions in such cases and is hard to visualize. Nevertheless, it is tempting to assume that the physical bounds are still spherical, albeit with an extension to higher dimensions. However, this turns out to be incorrect, in general. The physical bounds in some of the dimensions of Liouville space turn out not to be spheres but *regular simplexes*. A regular simplex in one dimension is a line, a regular simplex in two dimensions is an equilateral triangle, and a regular simplex in three dimensions is a regular tetrahedron, with the concept extending to arbitrary dimensions. In general, a simplex is the simplest possible convex object, where the term *convex* means that any two points belonging to the object may be connected by a straight line which never leaves the object. In general, a simplex in N dimensions is called an N -simplex, although some simplexes also have special names, such as the line (1-simplex), the triangle (2-simplex), the tetrahedron (3-simplex), and the *pentachoron* or *5-cell* (4-simplex) [Bibr bib1.bibx30].

The physical boundary of Liouville space is of little consequence for “conventional” NMR experiments, which are performed at or near thermal equilibrium. Under ordinary temperatures and magnetic fields, this is a region very close to the origin of Liouville space (except for the fixed projection onto the unity operator) and hence very far from the boundary. However, hyperpolarization techniques such as optical pumping [Bibr bib1.bibx43], dynamic nuclear polarization [Bibr bib1.bibx38], quantum-rotor-induced polarization [Bibr bib1.bibx39] and parahydrogen-induced polarization [Bibr bib1.bibx22] have provided ready access to regions which are “close to the edge”. Furthermore, spin systems which are in a highly non-equilibrium state are of great practical importance because of the greatly enhanced NMR signals that they can produce. The position and shape of the physical boundary have therefore become relevant.

Furthermore, some familiar concepts in magnetic resonance which were originally developed in the context of near-equilibrium spin dynamics do not retain validity far from the origin. An important case is the inhomogeneous master equation [Bibr bib1.bibx34], which fails close to the physical boundary of Liouville space, where it should be replaced by a Lindbladian master equation [Bibr bib1.bibx13].

## 3 Polarization moments

### 3.1 Isolated spins- I

For an ensemble of isolated spins- I , a suitable expansion of the form in Eq. ([Disp-formula Ch1.E2]) is as follows:

9 $$\rho =\sum_{\lambda =0}^{2I}\;\sum_{\mu =-I}^{+I}\rho_{\lambda \mu }T_{\lambda \mu }.$$

Here $\rho_{\lambda \mu }$ are complex numbers which are called here *polarization moments*, following the usage in the atomic physics community [Bibr bib1.bibx24]. The operators $T_{\lambda \mu }$ are *normalized irreducible spherical tensor operators* (NISTOs). They are normalized over the spin- I Hilbert space:

10 $$(T_{\lambda \mu }|T_{\lambda \mu })=\text{Tr}\{T_{\lambda \mu }^{{}^{†}}T_{\lambda \mu }\}=1.$$

The normalized spherical tensor operators $T_{\lambda \mu }$ differ from the operators $T_{\lambda \mu }$ commonly used in NMR theory [Bibr bib1.bibx70] by a multiplicative factor.

A semantic objection may be raised over the use of the term *polarization moment* for the case λ=0 . The term *polarization* is generally taken to imply an anisotropic distribution of dipole moments (magnetic or electric). However, the rank-0 moment represents an isotropic distribution of spin angular momentum and hence is not a “polarization” in a conventional sense. While acknowledging that this is a reasonable objection, we contend that the extension of the term “polarization” to cover rank-0 terms is too convenient to be blocked by pedantry. A similar objection arises over the term *singlet polarization* for spin pairs, as discussed below.

For isolated spins- I , the low-rank normalized spherical tensor operators are as follows, for the case μ=0 :

11 $$\begin{array}{rl} T_{00} & =N_{0}^{I}I\\ T_{10} & =N_{1}^{I}I_{z}\\ T_{20} & =N_{2}^{I}\frac{1}{\sqrt{6}}(3{I_{z}}^{2}-I(I+1)I)\\ T_{30} & =N_{3}^{I}\frac{1}{\sqrt{10}}(5{I_{z}}^{3}+(1-3I(I+1))I_{z}) \end{array}$$

The normalization factors are as follows:

12 $$\begin{array}{rl} N_{0}^{I} & =(2I+1{)}^{-1/2}\\ N_{1}^{I} & =\{\frac{1}{3}I(I+1)(2I+1){\}}^{-1/2}\\ N_{2}^{I} & =\{\frac{1}{30}I(I+1)(2I-1)(2I+3)(2I+1){\}}^{-1/2}\\ N_{3}^{I} & =\{\frac{1}{70}I(I+1)(I-1)(I+2)(2I-1)\\ & \;\;\times (2I+3)(2I+1){\}}^{-1/2} \end{array}$$

These normalization factors depend on the spin quantum number I and the rank λ but are independent of the component index μ .

It follows from Eqs. ([Disp-formula Ch1.E2]) and ([Disp-formula Ch1.E10]) and the orthogonality of the NISTOs that any polarization moment may be derived from the density operator by a Liouville bracket operation:

13 $$\rho_{\lambda \mu }=(T_{\lambda \mu }|\rho )=\text{Tr}\{T_{\lambda \mu }^{{}^{†}}\rho \}.$$

The polarization moments have the following symmetry:

14 $$\rho_{\lambda \mu }=(-1{)}^{-\mu }\rho_{\lambda -\mu }^{*},$$

which follows from the hermiticity of the density operator and the symmetries of the spherical tensor operator components [Bibr bib1.bibx75].

For isolated spins- I , the condition Tr{ρ}=1 fixes the value of the rank-0 polarization moment:

15 $$\rho_{00}=(2I+1{)}^{-1/2}.$$

The rank-1 polarization moment $\rho_{10}$ is proportional to the z polarization of the spin- I ensemble as follows:

16 $$\rho_{10}=\{\frac{1}{3I}(I+1)(2I+1){\}}^{-1/2}\;p_{z}.$$

Similarly, the rank-1 polarization moments $\rho_{1\pm 1}$ are proportional to complex combinations of the transverse polarizations:

17 $$\rho_{1\pm 1}=\{\frac{2}{3I}(I+1)(2I+1){\}}^{-1/2}(\mp p_{x}+ip_{y}).$$

The relationship in Eq. ([Disp-formula Ch1.E16]) evaluates as follows for some common spin quantum numbers I :

18 $$\begin{array}{rl} \rho_{10}=2^{-1/2}\;p_{z}\;\; & \text{(}I=1/2\text{)}\\ \rho_{10}=2^{-1/2}\;p_{z}\;\; & \text{(}I=1\text{)}\\ \rho_{10}=\frac{3}{2}5^{-1/2}\;p_{z}\;\; & \text{(}I=3/2\text{)}\\ \rho_{10}=(\frac{5}{14}{)}^{1/2}\;p_{z}\;\; & \text{(}I=5/2\text{)} \end{array}$$

In atomic physics, finite moments with rank λ=1 are called *orientation*, while finite moments with rank λ=2 are called *alignment* [Bibr bib1.bibx6]. Although the term *orientation* is not generally used for this purpose in the magnetic resonance community, the term *alignment* is used to imply rank-2 multipole order, particularly in the context of solid-state NMR as applied to quadrupolar nuclei [Bibr bib1.bibx12]. For isolated spins- I , the terms *spin alignment* and *quadrupolar order* may be regarded as synonymous.

Multipole expansions of the spin density operator as in Eq. ([Disp-formula Ch1.E2]) have long been used in NMR. Extensive theoretical development was performed by Sanctuary, Bowden, Bain and co-workers [Bibr bib1.bibx62] and has been exploited to generate graphical representations of density operator evolution [Bibr bib1.bibx58]. One of the salient early examples of the multipole description is the treatment of quadrupolar relaxation by Bodenhausen and co-workers [Bibr bib1.bibx40]. In this elegant paper, the relaxation dynamics of quadrupolar nuclei outside the extreme narrowing limit is treated in terms of propagation in the space of spherical tensor operators, drawing fruitful parallels with the concepts of coherence transfer pathways [Bibr bib1.bibx15].

There are also techniques for determining the polarization moments of a spin ensemble *experimentally* at any point during a pulse sequence by combining the signals from many successive experiments multiplied by complex factors. This method has been called *spherical tensor analysis* [Bibr bib1.bibx74] and has been applied to the study of endofullerenes [Bibr bib1.bibx28].

### 3.2 Spin-1/2 pairs

The construction of spherical tensor operators for systems of coupled spins is a complicated affair. Extensive expositions of the technique have been given [Bibr bib1.bibx62]. In this article, the discussion of coupled spin systems is restricted to the simplest case, namely pairs of coupled spins-1/2. Since the dimension of Hilbert space is $N_{H}=4$ , the dimension of Liouville space is $N_{L}=16$ . This space includes six orthogonal zero-quantum operators, four of which are symmetric with respect to spin exchange and two of which are antisymmetric. The four symmetric μ=0 operators are as follows:

19 $$\begin{array}{rl} T_{00}^{0} & =\frac{1}{2}I,\\ T_{00}^{12} & =-2\times 3^{-1/2}I_{1}\cdot I_{2},\\ T_{10}^{+} & =2^{-1/2}(I_{1z}+I_{2z}),\\ T_{20}^{12} & =(2/3{)}^{1/2}(3I_{1z}I_{2z}-I_{1}\cdot I_{2}). \end{array}$$

Note that spin-1/2 pairs support two different spherical tensor operators with rank λ=0 , denoted $T_{00}^{0}$ and $T_{00}^{12}$ . The plus superscript in $T_{10}^{+}$ indicates that the operators $I_{1z}$ and $I_{2z}$ are combined with the same sign.

The operator $T_{00}^{0}$ is proportional to the unity operator. The corresponding polarization moment is fixed by the condition Tr{ρ}=1 to the value

20 $$\rho_{00}^{0}=\frac{1}{2}.$$

The operator $T_{00}^{12}$ is proportional to the scalar product of the two spin angular momenta. The corresponding polarization moment is given by

21 $$\rho_{00}^{12}=\frac{\sqrt{3}}{2}p_{S},$$

where $p_{S}$ is called the *singlet polarization* or *singlet order* and corresponds to the population imbalance between the singlet state and the triplet manifold, in the spin-pair ensemble. In many cases, the singlet polarization is protected against common relaxation mechanisms and exhibits an extended lifetime [Bibr bib1.bibx27].

Since the moments $\rho_{00}^{0}$ and $\rho_{00}^{12}$ have spherical rank λ=0 , they both represent isotropic distributions of the spin angular momenta. As before, we contend that the extension of the term “polarization” to cover rank-0 spherical moments is too convenient to ignore while accepting that opinions may differ on the wisdom of this approach.

The operator $T_{10}^{+}$ corresponds to a symmetric combination of the z -angular momentum operators for the two spins. The corresponding polarization moment is proportional to the mean z polarization of the spin ensemble:

22 $$\rho_{10}^{+}=\frac{1}{\sqrt{2}}p_{z}.$$

The operator $T_{20}^{12}$ corresponds to the rank-2 spherical tensor operator of the coupled spin pair. The corresponding polarization moment $\rho_{20}^{12}$ is proportional to the rank-2 order (dipolar order) of the spin-pair ensemble.

## 4 Physical bounds of Liouville space

The population of each individual spin state is bounded by 0 and 1 , while the sum of all populations is equal to 1. These properties constrain the physically realizable values of the polarization moments.

The spin density operator of the isolated spin- I ensemble may be expressed as a superposition of spherical tensor operators, with rank λ taking values between 0 and 2I . The rank-0 moment is fixed to the value $\rho_{00}=(2I+1{)}^{-1}$ (Eq. [Disp-formula Ch1.E15]). From Eq. ([Disp-formula Ch1.E2]) and the orthonormality of the NISTOs (Eq. [Disp-formula Ch1.E10]), the population of the state $|I,M_{I}\rangle$ may be written as follows:

23 $$\langle I,M_{I}|\rho |I,M_{I}\rangle =\sum_{\lambda =0}^{2I}\langle I,M_{I}|T_{\lambda 0}|I,M_{I}\rangle \rho_{\lambda 0}.$$

Hence, for isolated spins- I , there exists a system of 2I+1 simultaneous inequalities on the μ=0 polarization moments:

24 $$0\;\leq \;\sum_{\lambda =0}^{2I}\;\langle I,M_{I}|T_{\lambda 0}|I,M_{I}\rangle \rho_{\lambda 0}\;\leq \;1,$$

for $M_{I}\in \{+I,+I-1\ldots -I\}$ . Together with Eq. ([Disp-formula Ch1.E15]), the system of inequalities in Eq. ([Disp-formula Ch1.E24]) defines the physical bounds of the μ=0 polarization moments.

The consequences are now explored for some common spin systems.

### 4.1 Spins-1/2

For isolated spins-1/2, the rank-0 polarization moment is given from Eq. ([Disp-formula Ch1.E15]) by

25 $$\rho_{00}=2^{-1/2}\;\;\text{for}\;I=1/2.$$

Equations ([Disp-formula Ch1.E24]) and ([Disp-formula Ch1.E25]) lead to the following physical bounds for the rank-1 polarization moment:

26 $$-2^{-1/2}\;\leq \;\rho_{10}\;\leq \;+2^{-1/2}\;\;\text{for}\;I=1/2.$$

From Eq. ([Disp-formula Ch1.E16]), this corresponds to the expected bounds on the z polarization of the spin ensemble:

27 $$-1\;\leq \;p_{z}\;\leq \;+1,$$

which should come as no surprise. No spin system may have more than 100 % polarization.

### 4.2 Spins-1

The bounds on the polarization moments are more complicated for an ensemble of isolated spins-1. The rank-0 polarization moment is given through Eq. ([Disp-formula Ch1.E15]) by

28 $$\rho_{00}=3^{-1/2}\;\;\text{for}\;I=1.$$

The inequalities on the rank-1 and rank-2 polarization moments evaluate as follows:

29 $$\begin{array}{rl} 0\;\leq & \frac{1}{3}+\frac{1}{\sqrt{2}}\rho_{10}+\frac{1}{\sqrt{6}}\rho_{20}\;\leq \;1,\\ 0\;\leq & \frac{1}{3}-\sqrt{\frac{2}{3}}\rho_{20}\;\leq \;1,\\ 0\;\leq & \frac{1}{3}-\frac{1}{\sqrt{2}}\rho_{10}+\frac{1}{\sqrt{6}}\rho_{20}\;\leq \;1\;\;\text{for}\;I=1. \end{array}$$

The values of $\left\{\rho_{10},\rho_{20}\right\}$ which satisfy the inequalities in Eq. ([Disp-formula Ch1.E29]) lie within the shaded triangle in Fig. [Fig Ch1.F1]. The vertices of the triangle have coordinates $\left\{\rho_{10},\rho_{20}\right\}$ given by $\left\{\pm 2^{-1/2},6^{-1/2}\right\}$ and $\left\{0,-(2/3{)}^{1/2}\right\}$ ; each vertex corresponds to a pure-state density operator, in which only one state is populated. Similar triangular bounds have been identified in the mathematics literature [Bibr bib1.bibx44].

**Figure 1 Ch1.F1:**
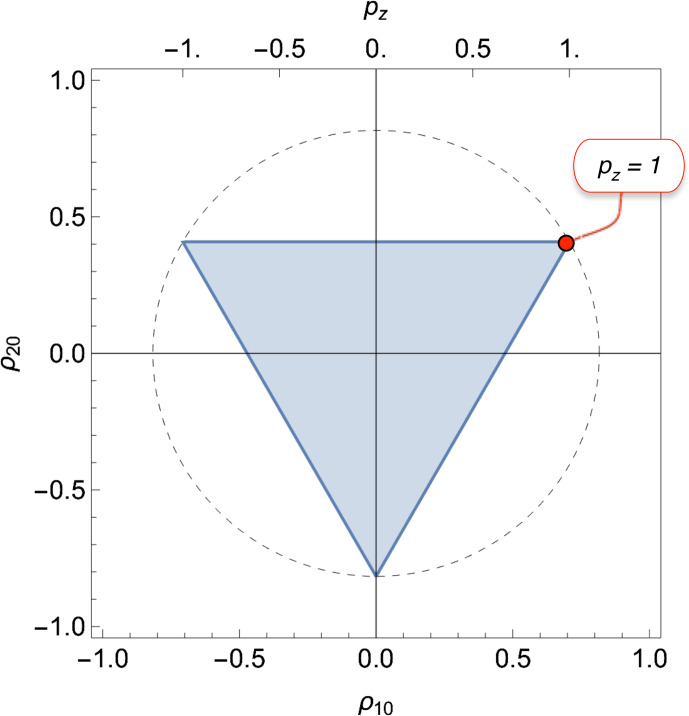
Physical bounds on the rank-1 and rank-2 polarization moments for isolated spins I=1 . The shaded triangle shows the physically accessible region. The vertices correspond to pure-state density operators for each of the three spin-1 Zeeman states. The radius of the dashed circle is $\sqrt{2/3}$ . The rank-1 polarization moment $\rho_{10}$ is related to the z polarization $p_{z}$ through Eq. ([Disp-formula Ch1.E18]). The red circle indicates a state with maximum Zeeman polarization.

The equilateral triangle in Fig. [Fig Ch1.F1] corresponds to a *regular simplex* in two dimensions.

The maximum z polarization of $p_{z}=1$ corresponds to the upper-right vertex. Figure [Fig Ch1.F1] shows that this highly polarized state corresponds to a mixture of rank-1 polarization (Zeeman order) and rank-2 polarization (quadrupolar order). It follows that the near-complete hyperpolarization of spin-1 nuclei, as performed by the Bodenhausen group [Bibr bib1.bibx3], generates hyperpolarized quadrupolar order as well as Zeeman order.

**Figure 2 Ch1.F2:**
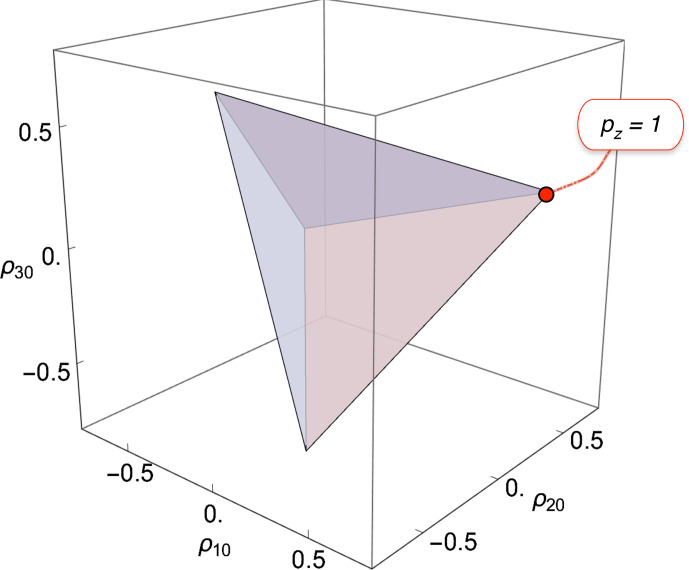
Physical bounds on the rank-1, rank-2, rank-3 polarization moments for isolated spins I=3/2 . The shaded tetrahedron shows the physically accessible region. The vertices correspond to pure-state density operators for each of the four spin-1 Zeeman states. The red circle indicates the position of maximum Zeeman polarization.

### 4.3 Spins-3/2

In the case of isolated spins-3/2, the rank-0 polarization moment is given by

30 $$\rho_{00}=4^{-1/2}=1/2\;\;\text{for}\;I=3/2.$$

For spins-3/2, there may be a finite rank-3 polarization moment $\rho_{30}$ as well as the rank-1 and rank-2 terms. The physical bounds on these polarization moments are set by the following inequalities:

31 $$\begin{array}{rl} 0\;\leq & \frac{1}{20}(5+6\sqrt{5}\rho_{10}+10\rho_{20}+2\sqrt{5}\rho_{30})\;\leq \;1\\ 0\;\leq & \frac{1}{20}(5+2\sqrt{5}\rho_{10}-10\rho_{20}-6\sqrt{5}\rho_{30})\;\leq \;1\\ 0\;\leq & \frac{1}{20}(5-2\sqrt{5}\rho_{10}-10\rho_{20}+6\sqrt{5}\rho_{30})\;\leq \;1\\ 0\;\leq & \frac{1}{20}(5-6\sqrt{5}\rho_{10}+10\rho_{20}-2\sqrt{5}\rho_{30})\;\leq \;1\\ & \;\;\text{for}\;\;I=3/2. \end{array}$$

The physical bounds on the three polarization moments constrain the spin density operator to the interior of the regular tetrahedron shown in Fig. [Fig Ch1.F2]. The vertices of the tetrahedron are at coordinates $\left\{\rho_{10},\rho_{20},\rho_{30}\right\}$ given by $\left\{\pm 1/2\sqrt{5},-1/2,\mp 3/2\sqrt{5}\right\}$ and $\left\{\pm 3/2\sqrt{5},1/2,\pm 1/2\sqrt{5}\right\}$ . Each vertex corresponds to a pure-state density operator, in which only one state is populated. The z polarization is related to the rank-1 polarization moment $\rho_{10}$ through Eq. ([Disp-formula Ch1.E18]).

The tetrahedron in Fig. [Fig Ch1.F2] corresponds to a *regular simplex* in three dimensions.

### 4.4 Higher spins

The treatment above is readily extended to higher spins. For isolated spins- I , the bounding figure is given by a *regular simplex* in 2I dimensions. For example, the four-dimensional bounding simplex of the polarization moments for spin I=2 is called a *5-cell* or *pentachoron* [Bibr bib1.bibx30]; the five-dimensional bounding simplex of the polarization moments for spin I=5/2 is called a *5-simplex* or *hexateron* [Bibr bib1.bibx30], and so on. Regular high-dimensional polytopes have been exploited before in NMR, albeit in a different context [Bibr bib1.bibx59].

**Figure 3 Ch1.F3:**
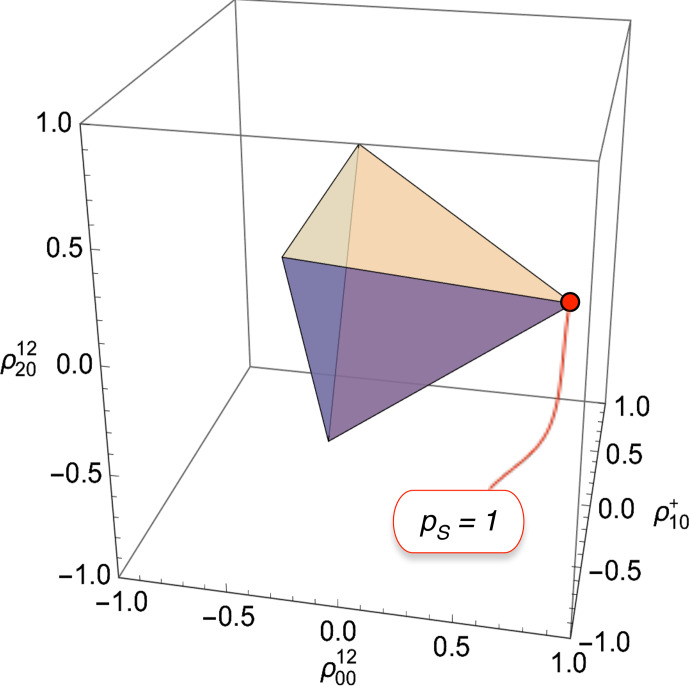
Physical bounds on the rank-0 polarization moment $\rho_{00}^{12}$ , the rank-1 polarization moment $\rho_{10}^{+}$ , and the rank-2 polarization moment $\rho_{20}^{12}$ for spin-1/2 pairs. The shaded tetrahedron shows the physically accessible region. The vertices correspond to pure-state density operators for each of the four singlet or triplet states. The annotation shows the vertex for exclusive population of the singlet state (corresponding to pure parahydrogen in the case of $H_{2}$ gas).

### 4.5 Spin-1/2 pairs

For spin-1/2 pairs, the symmetric μ=0 subspace is of dimension 4, spanned by the four symmetric spherical tensor operators given in Eq. ([Disp-formula Ch1.E19]). Since the polarization moment $\rho_{00}^{0}$ is fixed (Eq. [Disp-formula Ch1.E20]), the symmetric part of the spin density operator may be described as a point with coordinates $\left\{\rho_{00}^{12},\rho_{10}^{+},\rho_{20}^{12}\right\}$ , given by its projections onto the three orthonormal spherical tensor operators $\left\{T_{00}^{12},T_{10}^{+},T_{20}^{12}\right\}$ . The density operator may also include components that are antisymmetric with respect to exchange: these components lie outside this three-dimensional subspace and are not considered further here.

The physical bounds on the symmetrical polarization moments $\left\{\rho_{00}^{12},\rho_{10}^{+},\rho_{20}^{12}\right\}$ are set by the following inequalities:

32 $$\begin{array}{rl} 0\;\leq & \frac{1}{4}(1+2\sqrt{3}\rho_{00}^{12})\;\leq \;1\\ 0\;\leq & \frac{1}{12}(3-2\sqrt{3}\rho_{00}^{12}+6\sqrt{2}\rho_{10}^{+}+2\sqrt{6}\rho_{20}^{12})\;\leq \;1\\ 0\;\leq & \frac{1}{12}(3-2\sqrt{3}\rho_{00}^{12}-4\sqrt{6}\rho_{20}^{12})\;\leq \;1\\ 0\;\leq & \frac{1}{12}(3-2\sqrt{3}\rho_{00}^{12}-6\sqrt{2}\rho_{10}^{+}+2\sqrt{6}\rho_{20}^{12})\;\leq \;1\\ & \;\;\text{for}\;\text{spin-1/2}\;\text{pairs}. \end{array}$$

These inequalities constrain the polarization moments to the interior of the regular tetrahedron shown in Fig. [Fig Ch1.F3]. The vertices of the tetrahedron are at coordinates $\left\{\rho_{00}^{12},\rho_{10}^{+},\rho_{20}^{12}\right\}$ given by

33 $$\{\rho_{00}^{12},\rho_{10}^{+},\rho_{20}^{12}\}=\left\{\begin{array}{l} \left\{\frac{1}{2}3^{1/2},0,0\right\}\\ \left\{-\frac{1}{2}3^{-1/2},2^{-1/2},6^{-1/2}\right\}\\ \left\{-\frac{1}{2}3^{-1/2},-2^{-1/2},6^{-1/2}\right\}\\ \left\{-\frac{1}{2}3^{-1/2},0,-(2/3{)}^{1/2}\right\} \end{array}\right.$$

Each vertex corresponds to a pure-state density operator, in which the singlet state or one of the three triplet states is exclusively populated.

The highlighted point in Fig. [Fig Ch1.F3] has coordinates $\left\{\frac{1}{2}3^{1/2},0,0\right\}$ . From Eq. ([Disp-formula Ch1.E21]), this point corresponds to unit singlet polarization ( $p_{S}=1$ ) and hence a pure singlet density operator:

34 $$\rho =|S_{0}\rangle \langle S_{0}|,$$

where the singlet state is given by [Bibr bib1.bibx50]

35 $$|S_{0}\rangle =\frac{1}{\sqrt{2}}(|\alpha \beta \rangle -|\beta \alpha \rangle )$$

**Figure 4 Ch1.F4:**
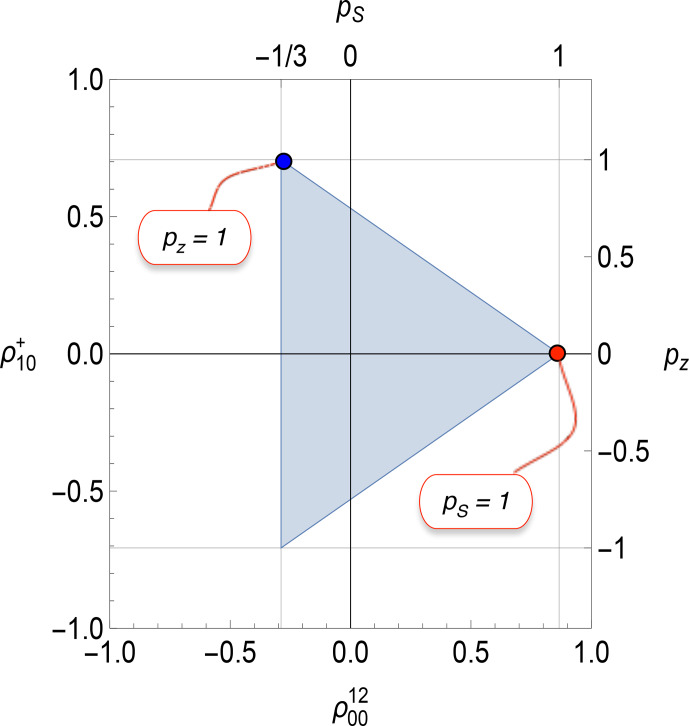
Physical bounds on the rank-0 polarization moment $\rho_{00}^{12}$ and the rank-1 polarization moment $\rho_{10}^{+}$ for spin-1/2 pairs. The corresponding values of the z polarization $p_{z}$ and singlet polarization $p_{S}$ are also shown. The singlet polarization $p_{S}$ (top horizontal axis) and rank-0 polarization moment $\rho_{00}^{12}$ (lower horizontal axis) are related through Eq. ([Disp-formula Ch1.E21]). The shaded triangle shows the physically accessible region. The annotations show the vertices for exclusive population of the singlet state (red) and for maximal z polarization (blue). Note that complete z polarization is accompanied by singlet polarization of $p_{S}=-1/3$ .

A projection of the tetrahedral bound in Fig. [Fig Ch1.F3] onto the $\left\{\rho_{00}^{12},\rho_{10}^{+}\right\}$ plane is shown in Fig. [Fig Ch1.F4]. The corresponding values of the singlet polarization $p_{S}$ and z polarization $p_{z}$ are shown on the axes, with the conversion factors given in Eqs. ([Disp-formula Ch1.E21]) and ([Disp-formula Ch1.E22]). The red point in Fig. [Fig Ch1.F4] shows that maximal singlet polarization is necessarily accompanied by zero z polarization. In the case that the spin-1/2 pair is composed of the two proton nuclei of $H_{2}$ , the red point corresponds to the spin density operator of pure parahydrogen [Bibr bib1.bibx35].

The blue point in Fig. [Fig Ch1.F4] shows that maximal z polarization is necessarily accompanied by singlet polarization of $p_{S}=-1/3$ . This reflects the fact that maximal z polarization can only be achieved by depleting the singlet state at the expense of one of the triplet states. This fact may be exploited experimentally to generate hyperpolarized (negative) long-lived singlet order by the application of low-temperature dynamic nuclear polarization to spin-pair systems [Bibr bib1.bibx73]. Analogous phenomena are observed in more complex spin systems, such as methyl groups [Bibr bib1.bibx33] and deuterated moieties [Bibr bib1.bibx45].

The physical bounds depicted in Figs. [Fig Ch1.F3] and [Fig Ch1.F4] are an intrinsic property of the spin-pair density operator and are completely independent of the spin Hamiltonian and its symmetry properties. Hence, these bounds apply to both magnetically equivalent and magnetically inequivalent spin-1/2 pairs. Nevertheless, since the spin Hamiltonian of magnetically inequivalent spin-1/2 pairs lacks exchange symmetry, it is also true that the density operator of magnetically inequivalent pairs readily accesses dimensions of Liouville space which are not exchange-symmetric and which are not included in these pictures. Hence, although Figs. [Fig Ch1.F3] and [Fig Ch1.F4] are equally valid for magnetically equivalent and inequivalent systems, there are additional dimensions which are not represented in these pictures and which are particularly relevant for the magnetically inequivalent case.

The geometry of the physical bounds is independent of the operator basis. Although a spherical tensor operator basis has been used in the discussion above, bounds of the same form are generated in any orthonormal operator basis, albeit with an overall rotation that depends on the relationship of the two bases. For example, if the ket–bra operator products |i〉〈j| are used as the basis of Liouville space, where $i,j\in \{1\ldots N_{H}\}$ , then the $N_{H}$ vertices of the bounding simplex are located at coordinates {1,0,0…} , {0,1,0,0…} , {0,0,1,0,…} , etc., representing the pure-state density operators with exclusive population of a single Hilbert-space state.

**Figure 5 Ch1.F5:**
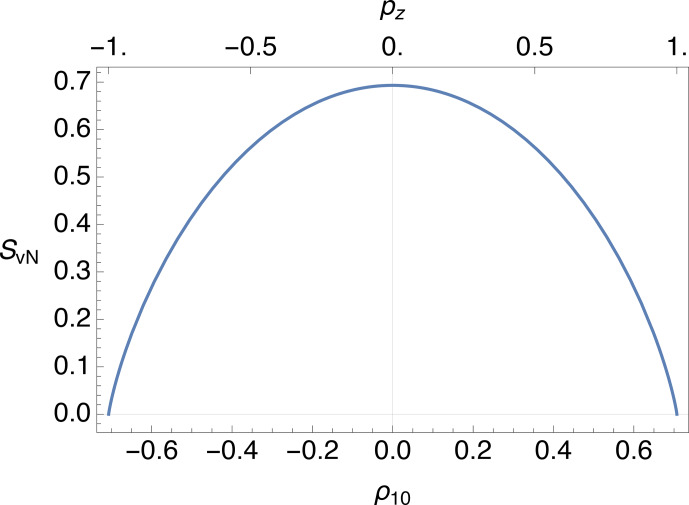
von Neumann entropy $S_{\mathrm{vN}}$ plotted against the rank-1 polarization moment $\rho_{10}$ and the z polarization $p_{z}$ for isolated spins-1/2, for the case of zero transverse polarizations $p_{x}=p_{y}=0$ . The maximum value of $S_{\mathrm{vN}}$ is ln⁡2≃0.693 .

## 5 von Neumann entropy

Quantum statistical mechanics uses the *von Neumann entropy* (vNE) to describe the disorder in, or absence of information about, a quantum system [Bibr bib1.bibx23]. It is derived from the spin density operator as follows:

36 $$S_{\mathrm{vN}}=-\text{Tr}\{\rho \mathrm{ln}\rho \}.$$

The vNE for a system in a pure quantum state is zero, while the vNE for a system with equal populations of $N_{H}$ quantum states, and no coherences, is given by $S_{\mathrm{vN}}=\mathrm{ln}N_{H}$ .

### 5.1 Spins-1/2

The von Neumann entropy $S_{\mathrm{vN}}$ is plotted against the rank-1 polarization moment $\rho_{10}$ in Fig. [Fig Ch1.F5], assuming that $\rho_{1\mu }=0$ for μ=±1 . The corresponding value of the z polarization $p_{z}=\sqrt{2}\rho_{10}$ is shown on the top margin of the plot. The entropy goes to zero for complete z polarization in the positive or negative sense ( $p_{z}=\pm 1$ ) and attains the maximum value of $S_{\mathrm{vN}}=\mathrm{ln}2$ for zero polarization. The maximum entropy of ln⁡2 reflects the equal populations of the two Zeeman eigenstates and absence of coherences, for a completely saturated system.

**Figure 6 Ch1.F6:**
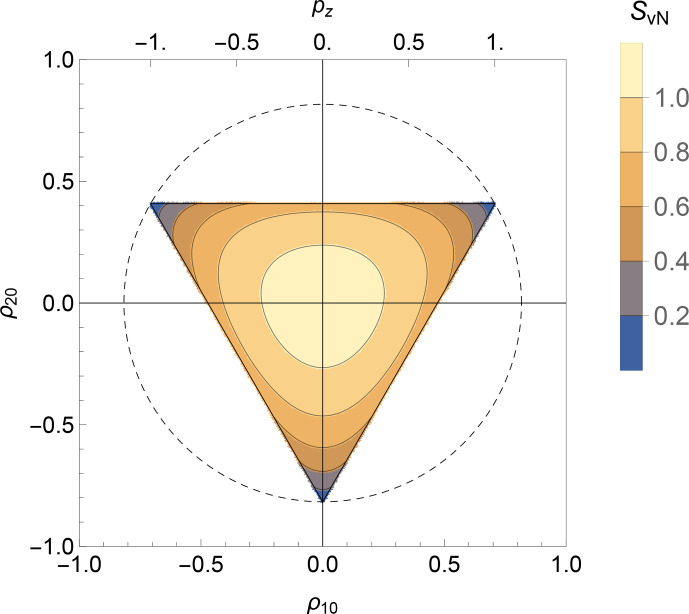
The von Neumann entropy $S_{\mathrm{vN}}$ is shown as a contour plot against the rank-1 and rank-2 polarization moments for isolated spins-1, for the case of $\rho_{\lambda \mu }=0$ for μ≠0 . Only the physical region is shown (see Fig. [Fig Ch1.F1]). The corresponding value of the z polarization $p_{z}=2^{1/2}\rho_{10}$ is shown along the top edge. The maximum value of the von Neumann entropy, reached at the origin, is ln⁡3≃1.10 .

### 5.2 Spins-1

For isolated spins-1, the von Neumann entropy is a function of the rank-1 and rank-2 polarization moments, assuming that all polarization moments $\rho_{\lambda \mu }$ vanish for μ≠0 . Figure [Fig Ch1.F6] shows a contour plot of the von Neumann entropy against $\rho_{10}$ and $\rho_{20}$ , assuming that all polarization moments with μ≠0 vanish. Only the physically allowed region is shown, delineated by the triangle, as in Fig. [Fig Ch1.F1]. The entropy goes to zero at the three vertices, which correspond to the pure-state density operators with 100 % population of a single state. The von Neumann entropy reaches the maximum value of ln⁡3 at the centre of the plot, corresponding to $\rho_{10}=\rho_{20}=0$ . The value of ln⁡3 reflects the equal distribution of population over the three spin states.

### 5.3 Higher spins

The behaviour of the von Neumann entropy is readily anticipated for higher spin quantum numbers. The entropy vanishes at the (2I+1) vertices of the 2I -simplex which bounds the physical region. The entropy maximum of ln⁡(2I+1) is reached at the origin of the space, which corresponds to equal populations for all of the 2I+1 spin states.

**Figure 7 Ch1.F7:**
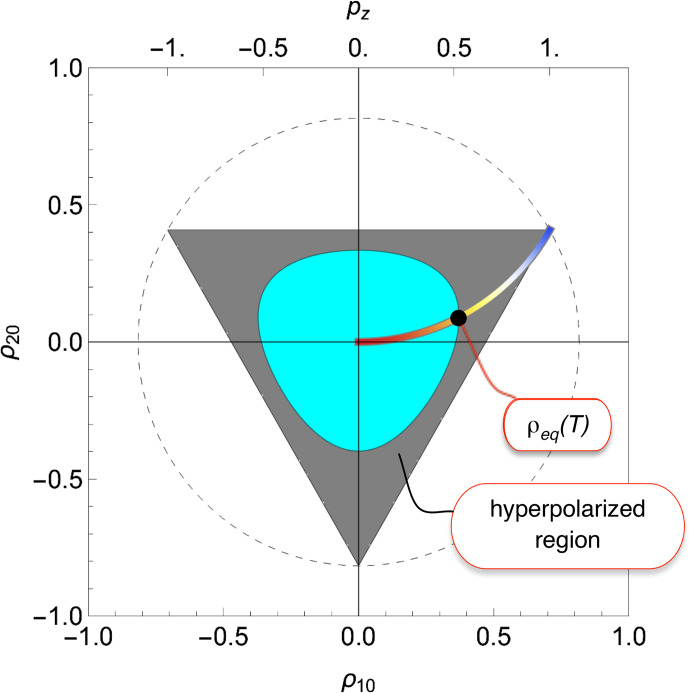
The coloured arc shows the set of thermal equilibrium density operators for spins-1 subjected to a dominant magnetic field. The spin temperature is indicated by colour, progressing from high (red) to low (blue). The black dot indicates the thermal equilibrium density operator at a particular temperature T . All points within the dark grey region represent hyperpolarized states of the spin-1 ensemble (density operators) at temperature T .

## 6 Hyperpolarization

### 6.1 Thermal equilibrium

Thermal equilibrium with the environment at temperature T is reached when the density operator adopts the following form:

37 $$\rho_{\mathrm{eq}}(T)=\frac{\mathrm{exp}\{-H_{\mathrm{coh}}/k_{B}T\}}{\text{Tr}\{\mathrm{exp}\{-H_{\mathrm{coh}}/k_{B}T\}\}},$$

where $H_{\mathrm{coh}}$ is the coherent part of the spin Hamiltonian (excluding all fluctuating terms which drive dissipation). Equation ([Disp-formula Ch1.E37]) describes a Boltzmann distribution of spin-state populations under the coherent Hamiltonian $H_{\mathrm{coh}}$ .

In many cases, the coherent Hamiltonian is dominated by the Zeeman interaction with the main magnetic field, $H_{\mathrm{coh}}≃\omega^{0}I_{z}$ , where the Larmor frequency is $\omega^{0}=-\gamma B^{0}$ , and $B^{0}$ is the magnetic field. In this case the thermal equilibrium density operator is given by

38 $$\rho_{\mathrm{eq}}≃\frac{\mathrm{exp}\{\beta I_{z}\}}{\text{Tr}\{\mathrm{exp}\{\beta I_{z}\}\}},$$

where the normalized inverse temperature is $\beta =-\omega^{0}/k_{B}T$ . Since Eq. ([Disp-formula Ch1.E38]) is non-linear in $I_{z}$ , the thermal equilibrium density operator contains high-rank polarization moments in thermal equilibrium.

The coloured arc in Fig. [Fig Ch1.F7] shows the set of thermal equilibrium density operators for a dominant Zeeman interaction (Eq. [Disp-formula Ch1.E38]), over a range of spin temperatures. Blue denotes a low spin temperature ( β→∞ ), while red denotes a high spin temperature ( β→0 ). Note the increase in the rank-2 polarization moment $\rho_{20}$ at low spin temperatures.

### 6.2 A criterion of hyperpolarization

The von Neumann entropy in thermal equilibrium at temperature T is given by

39 $$S_{\mathrm{vN}}^{\mathrm{eq}}(T)=-\text{Tr}\{\rho_{\mathrm{eq}}(T)\mathrm{ln}\rho_{\mathrm{eq}}(T)\},$$

where the thermal equilibrium density operator is given by Eq. ([Disp-formula Ch1.E37]). We propose the following criterion of hyperpolarization:

40 $$S_{\mathrm{vN}}<S_{\mathrm{vN}}^{\mathrm{eq}}(T)\;(\text{criterion of hyperpolarization}),$$

where T is the temperature of the environment. Note that this definition of hyperpolarization makes no explicit mention of population differences or the existence of a net magnetic moment in a certain direction.

The criterion in Eq. ([Disp-formula Ch1.E40]) identifies a region of Liouville space which is occupied by hyperpolarized states. For example, since the black dot in Fig. [Fig Ch1.F7] indicates the thermal equilibrium density operator for spins I=1 at temperature T , the contour line $S_{\mathrm{vN}}=S_{\mathrm{vN}}^{\mathrm{eq}}(T)$ delineates the region of hyperpolarization at temperature T . All density operators which are inside the dark grey region represent physically realizable hyperpolarized states of the spin ensemble.

Being inside the dark grey region is a sufficient but not necessary criterion of hyperpolarization. Points outside the dark grey region but within the pale blue region might also represent hyperpolarized states, in the case that polarization moments which are not represented in the diagram, i.e. $\rho_{\lambda \mu }$ with {λ,μ}≠{1,0} and {2,0} , are sufficiently large.

The criterion in Eq. ([Disp-formula Ch1.E40]) is readily applied to higher spin systems, including coupled spin systems. Under this definition, parahydrogen is hyperpolarized, since the corresponding density operator has a von Neumann entropy of zero, which is lower than that of any thermal equilibrium state at finite temperature, even though pure parahydrogen possesses no magnetic moment or net angular momentum in a given direction.

**Figure 8 Ch1.F8:**
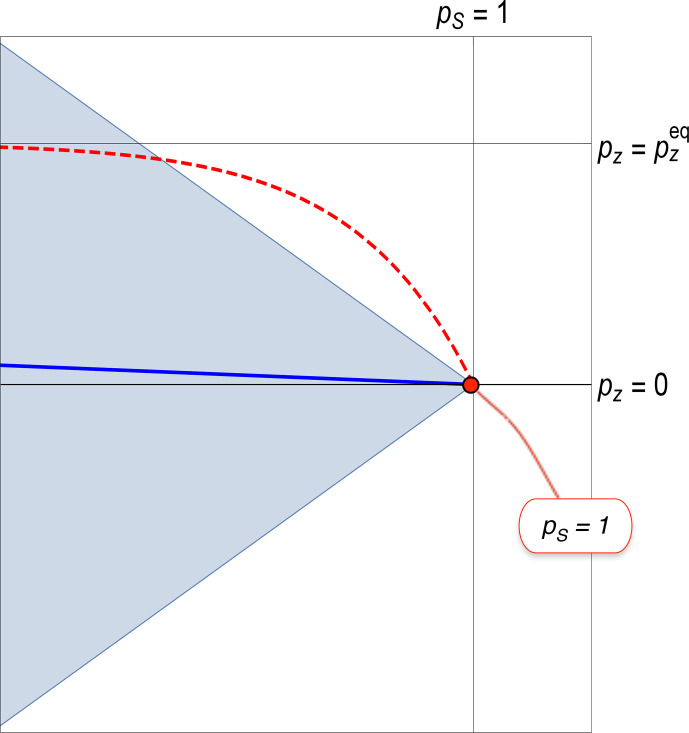
Non-equilibrium spin dynamics for an ensemble of spin-1/2 pairs, with $T_{S}\gg T_{1}$ , where $T_{S}$ is the rate constant for the decay of singlet order, and $T_{1}$ is the rate constant for the equilibration of z polarization. The plot shows an expanded view of Fig. [Fig Ch1.F4] in the vicinity of the red dot, which represents an initial state of 100 % singlet polarization. Dashed red line: trajectory predicted by the inhomogeneous master equation. Solid blue line: trajectory predicted by the Lindbladian master equation. Both trajectories eventually lead to the same thermal equilibrium state, represented by a point with coordinates $\left\{0,{p_{z}}^{\mathrm{eq}}\right\}$ , which is well beyond the left-hand edge of the plotted region and is not shown.

## 7 Non-equilibrium spin dynamics

The dynamics of the spin density operator is governed by a differential equation called the *master equation* which takes into account coherent influences on the spin system (such as external magnetic fields and non-fluctuating components of the spin interactions) as well as relaxation effects. Various forms of the master equation have been proposed. The most widely used form is called the *inhomogeneous master equation*, which has the following form:

41 $$\frac{d}{dt}\rho =-i{\hat{H}}_{\mathrm{coh}}\rho (t)+\hat{\Gamma }(\rho (t)-\rho_{\mathrm{eq}}),$$

where ${\hat{H}}_{\mathrm{coh}}$ is the commutation superoperator of the coherent Hamiltonian, and $\hat{\Gamma }$ is the relaxation superoperator [Bibr bib1.bibx60]. The equilibrium spin density operator $\rho_{\mathrm{eq}}$ is given by Eq. ([Disp-formula Ch1.E37]).

The inhomogeneous master Eq. ([Disp-formula Ch1.E41]) is valid for high-entropy states which are close to equilibrium and is a standard component of NMR theory [Bibr bib1.bibx60]. However, in a previous paper [Bibr bib1.bibx13], we showed that Eq. ([Disp-formula Ch1.E41]) loses validity for low-entropy states and may, in some cases, lead to non-physical predictions. We proposed a Lindbladian master equation, which has a wider range of validity.

This point is reinforced by Fig. [Fig Ch1.F8], which compares the predictions of the inhomogeneous and Lindbladian master equations when applied to spin-1/2 pairs in a low-entropy state of pure singlet polarization. The initial state of pure singlet order is shown by the red dot. The plot shows an expanded view of Liouville space, in the vicinity of the initial condition. The physical bounds of Liouville space are indicated by the blue triangle, as in Fig. [Fig Ch1.F4].

The red dashed line shows the trajectory predicted by the IME, in the case that $T_{S}\gg T_{1}$ , where $T_{S}$ is the relaxation time constant for singlet order [Bibr bib1.bibx50] and $T_{1}$ is the relaxation time constant for z polarization. Since $T_{1}$ is relatively short, the z polarization rapidly assumes its thermal equilibrium value $\rho_{z}^{\mathrm{eq}}$ , which is finite in the presence of a strong magnetic field. However, as shown in Fig. [Fig Ch1.F8], this leads the density operator into a forbidden region outside the physical boundary of Liouville space. This proves that the inhomogeneous master equation must be invalid in this regime.

The predicted trajectory of the Lindbladian master equation, as described in [Bibr bib1.bibx13], is shown by the blue line. This uneventful trajectory always stays well within the physical boundary of Liouville space.

## 8 Conclusions

This article has been an exploration of the geometry and physical boundary of Liouville space, the home territory of all spin density operators. In the past, most NMR experiments have only explored a tiny region of this space, very close to the origin (except for the fixed projection onto the unity operator). However, NMR experiments are increasingly performed on highly non-equilibrium spin states, which are sometimes located on or near the physical Liouville space boundary. We hope that this article is useful as a partial guide for wanderers in this region.

The word “partial” is used deliberately. So far, we have concentrated on the aspects of Liouville space which concern populations, and in the case of spin-1/2 pairs, on operators that are exchange-symmetric. The map still needs to be completed by delineating the physical bounds on coherences and on operators for multiple-spin systems, including those that are not exchange-symmetric. There has already been significant progress in that direction [Bibr bib1.bibx37].

The physical bounds discussed in this article should not be confused with the bounds on the unitary transformations of density operators [Bibr bib1.bibx68], which may also be represented by convex polytopes [Bibr bib1.bibx46]. The relationship between these different geometric bounds is another topic for future research.

## Data Availability

No data sets were used in this article.

## Code availability

The software code for the graphics shown in this paper is available from the authors on reasonable request.

## References

[bib1.bibx1] Abragam A (1961). The Principles of Nuclear Magnetism.

[bib1.bibx2] Adams RW, Aguilar JA, Atkinson KD, Cowley MJ, Elliott PIP, Duckett SB, Green GGR, Khazal IG, Lopez-Serrano J, Williamson DC (2009). Reversible Interactions with Para-Hydrogen Enhance NMR Sensitivity by Polarization Transfer. Science.

[bib1.bibx3] Aghelnejad B, Marhabaie S, Baudin M, Bodenhausen G, Carnevale D (2020). Spin Thermometry: A Straightforward Measure of Millikelvin Deuterium Spin Temperatures Achieved by Dynamic Nuclear Polarization. J Phys Chem Lett.

[bib1.bibx4] Ahuja P, Sarkar R, Vasos PR, Bodenhausen G (2009). Diffusion Coefficients of Biomolecules Using Long-Lived Spin States. J Am Chem Soc.

[bib1.bibx5] Ardenkjaer-Larsen JH, Fridlund B, Gram A, Hansson G, Hansson L, Lerche MH, Servin R, Thaning M, Golman K (2003). Increase in Signal-to-Noise Ratio of 
>
 10,000 Times in Liquid-State NMR. P Natl Acad Sci USA.

[bib1.bibx6] Auzinsh M, Budker D, Rochester S (2014). Optically Polarized Atoms: Understanding Light-Atom Interactions.

[bib1.bibx7] Bain AD (1978). Modulation of NMR Spin Echoes in Coupled Systems. Chem Phys Lett.

[bib1.bibx8] Bain AD (1980). A Superspin Analysis of Two-Dimensional FT NMR Experiments. J Magn Reson.

[bib1.bibx9] Bain AD (1980). Superspin in NMR: Application to the ABX System. J Magn Reson.

[bib1.bibx10] Bain AD (1984). Coherence Levels and Coherence Pathways in NMR. A Simple Way to Design Phase Cycling Procedures. J Magn Reson.

[bib1.bibx11] Banwell CN, Primas H (1963). On the Analysis of High-Resolution Nuclear Magnetic Resonance Spectra. Mol Phys.

[bib1.bibx12] Batchelder LS (2007). eMagRes.

[bib1.bibx13] Bengs C, Levitt MH (2020). A Master Equation for Spin Systems Far from Equilibrium. J Magn Reson.

[bib1.bibx14] Bengtsson I, Zyczkowski K (2006). Geometry of Quantum States: An Introduction to Quantum Entanglement.

[bib1.bibx15] Bodenhausen G, Kogler H, Ernst RR (1984). Selection of Coherence-Transfer Pathways in NMR Pulse Experiments. J Magn Reson.

[bib1.bibx16] Bornet A, Ji X, Mammoli D, Vuichoud B, Milani J, Bodenhausen G, Jannin S (2014). Long-Lived States of Magnetically Equivalent Spins Populated by Dissolution-DNP and Revealed by Enzymatic Reactions. Chem Eur J.

[bib1.bibx17] Bowden GJ, Hutchison WD (1986). Tensor Operator Formalism for Multiple-Quantum NMR. 3. Spin-1 Nuclei with an Asymmetry Term in the Quadrupole Hamiltonian. J Magn Reson.

[bib1.bibx18] Bowden GJ, Hutchison WD (1986). Tensor Operator Formalism for Multiple-Quantum NMR. 1. Spin-1 Nuclei. J Magn Reson.

[bib1.bibx19] Bowden GJ, Hutchison WD (1987). Tensor Operator Formalism for Multiple-Quantum NMR. 4. Spin-32 Nuclei with an Asymmetry Term in the Quadrupole Hamiltonian. J Magn Reson.

[bib1.bibx20] Bowden GJ, Hutchison WD, Khachan J (1986). Tensor Operator Formalism for Multiple-Quantum NMR. 2. Spins 3 2,2, and 5 2 and General I. J Magn Reson.

[bib1.bibx21] Bowden GJ, Martin JPD, Separovic F (1990). Tensorial Sets for Coupled Pairs of Spin-1/2 Nuclei. Mol Phys.

[bib1.bibx22] Bowers CR, Weitekamp DP (1987). Parahydrogen and Synthesis Allow Dramatically Enhanced Nuclear Alignment. J Am Chem Soc.

[bib1.bibx23] Breuer H-P, Petruccione F (2010). The Theory of Open Quantum Systems.

[bib1.bibx24] Budker D, Gawlik W, Kimball DF, Rochester SM, Yashchuk VV, Weis A (2002). Resonant Nonlinear Magneto-Optical Effects in Atoms. Rev Mod Phys.

[bib1.bibx25] Byrd MS, Khaneja N (2003). Characterization of the Positivity of the Density Matrix in Terms of the Coherence Vector Representation. Phys Rev A.

[bib1.bibx26] Carravetta M, Levitt MH (2004). Long-Lived Nuclear Spin States in High-Field Solution NMR. J Am Chem Soc.

[bib1.bibx27] Carravetta M, Johannessen OG, Levitt MH (2004). Beyond the 
$T_{1}$
 Limit: Singlet Nuclear Spin States in Low Magnetic Fields. Phys Rev Lett.

[bib1.bibx28] Carravetta M, Danquigny A, Mamone S, Cuda F, Johannessen OG, Heinmaa I, Panesar K, Stern R, Grossel MC, Horsewill AJ, Samoson A, Murata M, Murata Y, Komatsu K, Levitt MH (2007). Solid-State NMR of Endohedral Hydrogen-Fullerene Complexes. Phys Chem Chem Phys.

[bib1.bibx29] Cavadini S, Dittmer J, Antonijevic S, Bodenhausen G (2005). Slow Diffusion by Singlet State NMR Spectroscopy. J Am Chem Soc.

[bib1.bibx30] Coxeter HSM (1963). Regular Polytopes.

[bib1.bibx31] Dumez J-N (2019). Perspective on Long-Lived Nuclear Spin States. Mol Phys.

[bib1.bibx32] Dumez J-N, Håkansson P, Mamone S, Meier B, Stevanato G, Hill-Cousins JT, Roy SS, Brown RCD, Pileio G, Levitt MH (2015). Theory of Long-Lived Nuclear Spin States in Methyl Groups and Quantum-Rotor Induced Polarisation. J Chem Phys.

[bib1.bibx33] Dumez J-N, Vuichoud B, Mammoli D, Bornet A, Pinon AC, Stevanato G, Meier B, Bodenhausen G, Jannin S, Levitt MH (2017). Dynamic Nuclear Polarization of Long-Lived Nuclear Spin States in Methyl Groups. J Phys Chem Lett.

[bib1.bibx34] Ernst RR, Bodenhausen G, Wokaun A (1987). Principles of Nuclear Magnetic Resonance in One and Two Dimensions.

[bib1.bibx35] Farkas A (1935). Orthohydrogen, Parahydrogen and Heavy Hydrogen.

[bib1.bibx36] Garon A, Zeier R, Glaser SJ (2015). Visualizing Operators of Coupled Spin Systems. Phys Rev A.

[bib1.bibx37] Goyal SK, Simon BN, Singh R, Simon S (2016). Geometry of the Generalized Bloch Sphere for Qutrits. J Phys A.

[bib1.bibx38] Griffin RG, Prisner TF (2010). High Field Dynamic Nuclear Polarization – the renaissance. Phys Chem Chem Phys.

[bib1.bibx39] Icker M, Berger S (2012). Unexpected Multiplet Patterns Induced by the Haupt-Effect. J Magn Reson.

[bib1.bibx40] Jaccard G, Wimperis S, Bodenhausen G (1986). Multiple-quantum NMR Spectroscopy of S=3/2 Spins in Isotropic Phase: A New Probe for Multiexponential Relaxation. J Chem Phys.

[bib1.bibx41] Jannin S, Bornet A, Melzi R, Bodenhausen G (2012). High Field Dynamic Nuclear Polarization at 6.7T: Carbon-13 Polarization above 70 % within 20 min. Chem Phys Lett.

[bib1.bibx42] Jeener J (1982). Superoperators in Magnetic Resonance. Advances in Magnetic and Optical Resonance.

[bib1.bibx43] Kastler A (1957). Optical Methods of Atomic Orientation and of Magnetic Resonance. J Opt Soc Am (JOSA).

[bib1.bibx44] Kimura G, Kossakowski A (2005). The Bloch-Vector Space for N-Level Systems: The Spherical-Coordinate Point of View. Open Syst Inf Dyn.

[bib1.bibx45] Kress T, Walrant A, Bodenhausen G, Kurzbach D (2019). Long-Lived States in Hyperpolarized Deuterated Methyl Groups Reveal Weak Binding of Small Molecules to Proteins. J Phys Chem Lett.

[bib1.bibx46] Levitt MH, Bagguley DMS (1992). Pulsed Magnetic Resonance: NMR, ESR and Optics. A Recognition of E. L. Hahn.

[bib1.bibx47] Levitt MH (1992). Unitary Evolution, Liouville Space and Local Spin Thermodynamics. J Magn Reson.

[bib1.bibx48] Levitt MH, Khetrapal CL, Kumar A, Ramanathan KV (2010). Future Directions of NMR.

[bib1.bibx49] Levitt MH (2016). Symmetry Constraints on Spin Dynamics: Application to Hyperpolarized NMR. J Magn Reson.

[bib1.bibx50] Levitt MH (2019). Long Live the Singlet State!. J Magn Reson.

[bib1.bibx51] Mammoli D, Vuichoud B, Bornet A, Milani J, Dumez J-N, Jannin S, Bodenhausen G (2015). Hyperpolarized Para-Ethanol. J Phys Chem B.

[bib1.bibx52] Mamone S, Pileio G, Levitt MH (2010). Orientational Sampling Schemes Based on Four Dimensional Polytopes. Symmetry.

[bib1.bibx53] Mehring M (1976). High Resolution NMR Spectroscopy in Solids, NMR Basic Principles and Progress.

[bib1.bibx54] Meier B, Dumez J-N, Stevanato G, Hill-Cousins JT, Roy SS, Håkansson P, Mamone S, Brown RCD, Pileio G, Levitt MH (2013). Long-Lived Nuclear Spin States in Methyl Groups and Quantum-Rotor-Induced Polarization. J Am Chem Soc.

[bib1.bibx55] Navon G, Song Y-Q, Rõõm T, Appelt S, Taylor RE, Pines A (1996). Enhancement of Solution NMR and MRI with Laser-Polarized Xenon. Science.

[bib1.bibx56] Nielsen NC, Sørensen OW (1995). Conditional Bounds on Polarization Transfer. J Magn Reson Ser A.

[bib1.bibx57] Pell AJ (2021). A Method to Calculate the NMR Spectra of Paramagnetic Species Using Thermalized Electronic Relaxation. J Magn Reson.

[bib1.bibx58] Philp DJ, Kuchel PW (2005). A Way of Visualizing NMR Experiments on Quadrupolar Nuclei. Concept Magnetic Res Part A.

[bib1.bibx59] Pileio G, Levitt MH (2008). Isotropic Filtering Using Polyhedral Phase Cycles: Application to Singlet State NMR. J Magn Reson.

[bib1.bibx60] Redfield AG (1965). The Theory of Relaxation Processes. Advances in Magnetic and Optical Resonance.

[bib1.bibx61] Rodin BA, Bengs C, Kiryutin AS, Sheberstov KF, Brown LJ, Brown RCD, Yurkovskaya AV, Ivanov KL, Levitt MH (2020). Algorithmic Cooling of Nuclear Spins Using Long-Lived Singlet Order. J Chem Phys.

[bib1.bibx62] Sanctuary BC (1976). Multipole Operators for an Arbitrary Number of Spins. J Chem Phys.

[bib1.bibx63] Sanctuary BC (1980). Magnetic Multipoles in Time Dependent Fields. J Chem Phys.

[bib1.bibx64] Sanctuary BC, Temme FP (1985). Multipole N.M.R.: XIII. Multispin Interactions and Symmetry in Liouville Space. Mol Phys.

[bib1.bibx65] Sanctuary BC, Temme FP (1985). Multipole NMR. Molecular Physics.

[bib1.bibx66] Sarkar R, Ahuja P, Moskau D, Vasos PR, Bodenhausen G (2007). Extending the Scope of Singlet-State Spectroscopy. ChemPhysChem.

[bib1.bibx67] Sarkar R, Vasos PR, Bodenhausen G (2007). Singlet-State Exchange NMR Spectroscopy for the Study of Very Slow Dynamic Processes. J Am Chem Soc.

[bib1.bibx68] Sørensen OW (1990). A Universal Bound on Spin Dynamics. J Magn Reson.

[bib1.bibx69] Sørensen OW, Eich GW, Levitt MH, Bodenhausen G, Ernst RR (1984). Product Operator Formalism for the Description of NMR Pulse Experiments. Prog Nucl Mag Res Sp.

[bib1.bibx70] Spiess HW, Steigel A, Spiess HW (1978). Dynamic NMR Spectroscopy, NMR Basic Principles and Progress/Grundlagen Und Fortschritte, 15.

[bib1.bibx71] Suzuki M, Kubo R (1964). Theoretical Calculation of NMR Spectral Line Shapes. Mol Phys.

[bib1.bibx72] Szymański K, Weis S, Życzkowski K (2018). Classification of Joint Numerical Ranges of Three Hermitian Matrices of Size Three. Linear Algebra Appl.

[bib1.bibx73] Tayler MCD, Marco-Rius I, Kettunen MI, Brindle KM, Levitt MH, Pileio G (2012). Direct Enhancement of Nuclear Singlet Order by Dynamic Nuclear Polarization. J Am Chem Soc.

[bib1.bibx74] van Beek JD, Carravetta M, Antonioli GC, Levitt MH (2005). Spherical Tensor Analysis of Nuclear Magnetic Resonance Signals. J Chem Phys.

[bib1.bibx75] Varshalovich DA, Moskalev AN, Kheronskii VK (1988). Quantum Theory of Angular Momentum.

